# Regulation of the Tumor-Suppressor BECLIN 1 by Distinct Ubiquitination Cascades

**DOI:** 10.3390/ijms18122541

**Published:** 2017-11-27

**Authors:** Fahd Boutouja, Rebecca Brinkmeier, Thomas Mastalski, Fouzi El Magraoui, Harald W. Platta

**Affiliations:** 1 Biochemie Intrazellulärer Transportprozesse, Ruhr-Universität Bochum, 44801 Bochum, Germany; fahd.boutouja@rub.de (F.B.); rebecca.brinkmeier@rub.de (R.B.); thomas.mastalski@rub.de (T.M.); 2 Biomedizinische Forschung, Leibniz-Institut für Analytische Wissenschaften-ISAS-e.V. 44139 Dortmund, Germany; fouzi.elmagraoui@isas.de

**Keywords:** BECLIN 1, VPS34, AMBRA 1, ubiquitin, autophagy, tumor suppressor

## Abstract

Autophagy contributes to cellular homeostasis through the degradation of various intracellular targets such as proteins, organelles and microbes. This relates autophagy to various diseases such as infections, neurodegenerative diseases and cancer. A central component of the autophagy machinery is the class III phosphatidylinositol 3-kinase (PI3K-III) complex, which generates the signaling lipid phosphatidylinositol 3-phosphate (PtdIns3P). The catalytic subunit of this complex is the lipid-kinase VPS34, which associates with the membrane-targeting factor VPS15 as well as the multivalent adaptor protein BECLIN 1. A growing list of regulatory proteins binds to BECLIN 1 and modulates the activity of the PI3K-III complex. Here we discuss the regulation of BECLIN 1 by several different types of ubiquitination, resulting in distinct polyubiquitin chain linkages catalyzed by a set of E3 ligases. This contribution is part of the Special Issue “Ubiquitin System”.

## 1. Formation and Function of the PI3K-III Complex

Macroautophagy, hereafter referred to as autophagy, is a cell-protective mechanism that is based on the degradation of intracellular targets within the lytic compartment of the cell, which is the lysosome in mammals and the vacuole in yeasts and plants. This enables the general recycling of macromolecules during starvation as well as the selective elimination of potential harmful structures, such as protein aggregates, damaged organelles or phagocytosed pathogens.

In general, autophagy functions as a cytoprotective mechanism under stress conditions. Therefore, it contributes to the protection against infections, neurodegenerative disorders and cancer. However, this also means that the cytoprotective function of autophagy can promote tumor growth and resistance to chemotherapy in already established tumor cells that had transformed via the malfunction of another mechanism. Therefore, autophagy is often attributed as “double-edged sword” [1,2,3].

The autophagy mechanism can be divided in four basic steps. The initiation phase (I), involves the recognition of potential stimuli, like nutrient sensing. The phase is controlled by the signalling cascades linked to the autophagy-inhibiting mTOR (*mechanistic target of rapamycin*) kinase complex or the autophagy-supporting ULK1 (*unc-51 like autophagy activating kinase 1*) kinase complex. In the nucleation phase (II), the intracellular targets begin to be engulfed by a phagophore membrane. The start of this phagophore formation requires the VPS34 (*vacuolar protein sorting 34*) lipid kinase complex, which generates phosphorylated signalling lipids that attract further factors required for this step. At the end of the elongation phase (III), the targets are completely engulfed in the autophagosome. This maturation of the autophagosome depends on two ubiquitin-like conjugation systems, namely ATG12 (*autophagy-related 12*) and LC3 (*1A/1B light chain 3*) (Atg8 in yeast), as well as on the VPS34 lipid kinase complex. Finally, the autophagosome fuses with the lysosome, depending on the small GTPase RAB7 (*ras-related in brain 7*), and forms the autolysosome, in which the targets are hydrolysed (IV) [4].

### 1.1. Principles of PtdIns3P-Signaling during Autophagy

One of the key factors of the autophagy machinery is VPS34, which is a phosphatidylinositol 3-kinase (PI3K). In general, three classes of PI3Ks have been defined in mammals, which are named PI3K class I, II and III. They have in common that they phosphorylate the 3-hydroxyl group of the inositol ring within the membrane lipid phosphatidylinositol (PtdIns), but differ in their substrate specificity and therefore generate distinct phosphoinositide-species [5,6,7]. PI3K class I uses PtdIns(4,5)P_2_ as substrate to generate PtdIns(3,4,5)P_3_, which, among other functions, suppresses autophagy and supports receptor down-regulation and cell growth. PI3K class II uses PtdIns4P as substrate to generate PtdIns(3,4)P_2_, which is involved in receptor signaling and endocytosis [7,8]. The most conserved PI3K is the multi-subunit kinase complex PI3K-III. The catalytic subunit is VPS34, which only uses PtdIns as substrate in order to generate PtdIns3P [9,10,11]. Moreover, it is the only PI3K in yeast and plants [12,13,14].

PtdIns3P has been detected at endosomes, multivesicular bodies [15], phagosomes [16,17], midbodies [18], peroxisomes [19] and omegasomes [20,21,22]. Downstream effector proteins are recruited to PtdIns3P-enriched membrane domains. These PtdIns3P effector proteins are characterized by a corresponding binding motif, like the FYVE (*Fab1p*, *YOTB*, *Vac1p* and *EEA1*) domain or the PX (*Phox homology*) domain [23].

The subsequent PtdIns3P-dependent signaling cascades regulate not only autophagy, but can also be involved in other downstream events such as the down-regulation of growth factor receptors, endocytic signaling or cytokinesis in a context-dependent manner [22,24,25,26,27].

### 1.2. The Composition of the PI3K-III Complex

**Figure 1:**
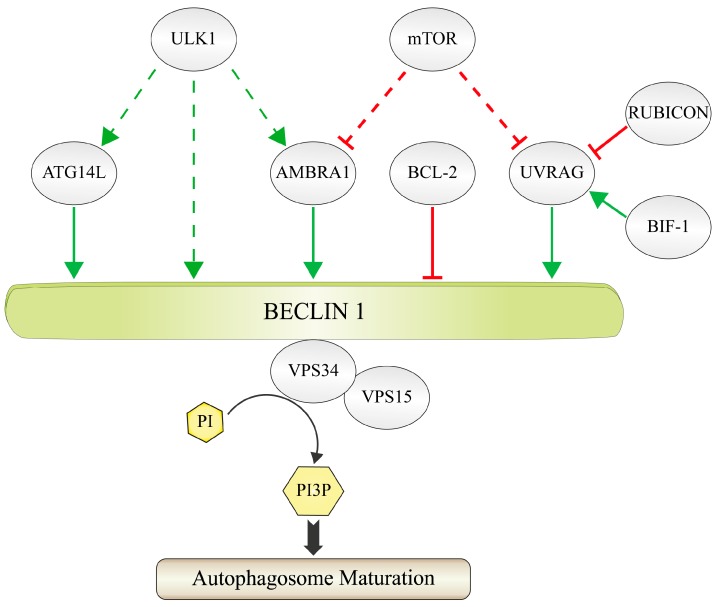
Composition of the PI3K-III complex. The figure shows the most important constituents of the PI3K-III complex in mammals. VPS34 is the catalytic subunit, which uses phosphatidylinositol (PI) as substrate in order to generate phosphatidylinositol 3-phosphate (PI3P). VPS34 forms the core complex together with the putative protein kinase VPS15 and the adaptor protein BECLIN 1. Additional regulatory proteins can bind to the core complex via BECLIN 1 in a context-dependent manner and therefore may not all be present at the same time. ATG14L, AMBRA 1 and UVRAG support the activity of VPS34 and therefore autophagosome maturation (green arrows). The contribution of UVRAG is supported by BIF-1 and inhibited by RUBICON (red lines). BCL-2 is an inhibitor of BECLIN 1. AMBRA 1 and UVRAG are inactivated via the phosphorylation by the protein kinase mTOR (red dashed lines). AMBRA 1, ATG14L and BECLIN 1 are activated via the phosphorylation by the protein kinase ULK1 (green dashed arrows).

In order to gain full enzymatic and biological activity, VPS34 has to associate with additional regulatory factors (Figure 1). The core components of the PI3K-III complex are the putative protein kinase VPS15 (also called p150) and the multivalent adaptor protein BECLIN 1 (*BECN1: coiled-coil, moesin-like BCL2-interacting protein*), which is called Atg6 or Vps30 in yeasts and plants [13,24,28]. The myristoylated VPS15 associates VPS34 with membranes via the interaction of its protein kinase domain to VPS34 [12,29]. BECLIN 1 interacts with an increasing number of transiently associated accessory factors. This dynamic subunit compositions specifies the localization, activity and physiological context of VPS34-catalyzed PtdIns3P production [24]. As a consequence, BECLIN 1 itself is tightly regulated by competing interaction partners, different subcellular localizations [30], phosphorylation [31] as well as ubiquitination [32,33,34,35]. This review presents an update on the growing number of ubiquitin-based regulatory mechanisms concerning BECLIN 1 (see Section 2.1, Section 2.2, Section 2.3, Section 2.4, Section 2.5, Section 2.6, Section 2.7, Section 2.8, Section 2.9, Section 2.10, and Section 2.11).

Two major versions of the PI3K-III complex have been described, which differ in the presence of additional complex constituents. ATG14L (*autophagy-related 14-like*) (also called BARKOR) and UVRAG (*ultraviolet irradiation resistance-associated gene*) are the mutual exclusive constituents of the two distinct PI3K-III complexes [36,37,38,39].

The ATG14L-containing PI3K-III complex plays a major role in autophagy [36,38,39]. ATG14L is required for the targeting of the PI3K-III complex to the endoplasmic reticulum (ER) [40], where it interacts with Syntaxin 17 at ER-mitochondria contact sites [41], which are discussed as one possible source for autophagosomal membranes [42]. Furthermore, the interaction of ATG14L with BECLIN 1 controls the accessibility of certain amino acid residues within BECLIN 1 that are target sites for the phosphorylation of BECLIN 1 [43].

ATG14L has also autophagy-independent functions. It can interact with the SNARE-associated protein Snapin and thereby contributes to the coordination of endosome maturation and endocytic trafficking [44].

Another constituent of the autophagy-related PI3K-III complex is AMBRA 1 (*activating molecule in BECLIN 1-regulated autophagy*) [45]. AMBRA 1 is a WD40-protein that binds to BECLIN 1. It supports PtdIns3P production via the stabilization of the VPS34-BECLIN 1 interaction [45]. AMBRA 1 regulates autophagy also via additional mechanisms. AMBRA 1 binds to the dynein light chain 1 (DLC1) at the dynein motor complex, where it also concentrates BECLIN 1 [46]. Subsequent to the initiation of autophagy, AMBRA 1 gets phosphorylated by ULK1. The phosphorylation leads to the dissociation of AMBRA 1 from the motor complex and enables the transport of AMBRA 1 to the ER. Here, AMBRA 1 can contribute to autophagosome formation [46]. In addition, AMBRA 1 plays a central role in several ubiquitin-dependent steps in the regulation of different aspects of autophagy, as will be discussed later (see Section 2.1 and Section 2.3). AMBRA 1 seems to have autophagy-independent functions as it is linked to apoptosis and cell cycle control [47].

The UVRAG-containing PI3K-III complex is involved in membrane trafficking events and contributes both to phagophore maturation as well as endosome maturation [38,39]. These seemingly distinct roles converge in order to enhance the delivery of cargos to the lysosome. The BECLIN 1-bound UVRAG associates with the class C VPS complex at endosomes, where it stimulates the GTPase activity of RAB7 [48]. This enables a higher rate of fusion events between autophagosomes and late endosome/lysosomes, which finally results in an accelerated delivery and degradation of autophagic cargo. Moreover, the class C VPS- bound UVRAG stimulates endosome–endosome fusion events as well, which leads to a rapid degradation of endocytic cargo [48]. 

The UVRAG-associated PI3K-III complex component BIF-1 (*endophilin B1/BAX-interacting factor 1*) supports membrane trafficking events by enhancing VPS34 kinase activity and PtdIns3P-production [49] in addition to its membrane sculpturing abilities [50,51]. In addition, BIF-1 contributes to the endocytic degradation of NGF (*nerve growth factor*) [52] and EGF (*epidermal growth factor*) [53].

Two major negative regulators of the PI3K-III complexes have been described. One is the UVRAG- and BECLIN 1-associated protein RUBICON (*run domain beclin-1-interacting and cysteine-rich domain-containing protein*), which inhibits VPS34 activity [38,39,54]. RUBICON binds to the PI3K-III complex when BECLIN 1 is acetylated by p300 [55], which results in a block of endocytic trafficking and autophagosome maturation.

The other important inhibitor of the PI3K-III function is the anti-apoptotic proto-oncogene BCL-2 (*B-cell lymphoma 2*) [56,57,58]. BCL-2 binds to the BH3 (*Bcl-2-homolgy-3*)-domain of BECLIN 1 [57,59]. The association of BECLIN 1 with BCL-2 is thought to prevent the interaction of BECLIN 1 with factors required for the formation of a functional VPS34 complex, which results in a block of autophagy [60,61,62,63]. The interaction of BCL-2 with BECLIN 1 is regulated by several mechanisms. This involves the competition with additional binding partners, which blocks the interaction of BCL-2 and BECLIN 1 [64], as well as the block of this interaction by phosphorylation [31,35] or ubiquitination (see Section 2.3).

## 2. Regulation of BECLIN 1 via Distinct Ubiquitination Machineries

BECLIN 1 is of central importance for the activity of the PI3K-III complex. It functions as the central adapter module within the PI3K-III complex, because it interacts with most of the additional binding partners [30,65,66]. Therefore, several factors of the PI3K-III complex regulation target BECLIN 1. Accumulating evidence in recent years shows that BECLIN 1 is the substrate of versatile ubiquitination machineries that modify this central protein with distinct ubiquitin signals.

### 2.1. Lys63-Linked Polyubiquitination of BECLIN 1 by the AMBRA 1-Containing CUL4-Ligase Complex

The activity of the PI3K-III complex is regulated by AMBRA 1 in different ways. The interaction of AMBRA 1 with BECLIN 1 stabilizes the VPS34-BECLIN 1 interaction [45], suggesting an allosteric effect. Moreover, AMBRA 1 binds to the dynein light chain 1 and is involved in the assembly of the PI3K-III complex at the ER, where it contributes to the process of autophagosome formation [46]. Another mode of regulation concerns the influence of AMBRA 1 on posttranslational modification of BECLIN 1 with ubiquitin [67].

AMBRA 1 associates with a multi-subunit ligase of the CULLIN-RING (CR)-family. This complex contains the RING (*really interesting new gene*)-type ubiquitin-protein isopeptide ligase (E3 ligase [68]) RBX1 (*ring-box 1*) as catalytic subunit, the scaffold protein CUL4 (*cullin 4*) as well as different substrate-selective adaptors [69]. AMBRA 1 was demonstrated to be a CUL4-associated protein, which is indicated by an alias of AMBRA 1, DCAF3 (*DDB1- and CUL4-associated factor 3*) [70,71]. In general, DCAF3 substrate receptors like AMBRA 1 confer the substrate specificity of complex E3 ligases [71,72].

**Figure 2:**
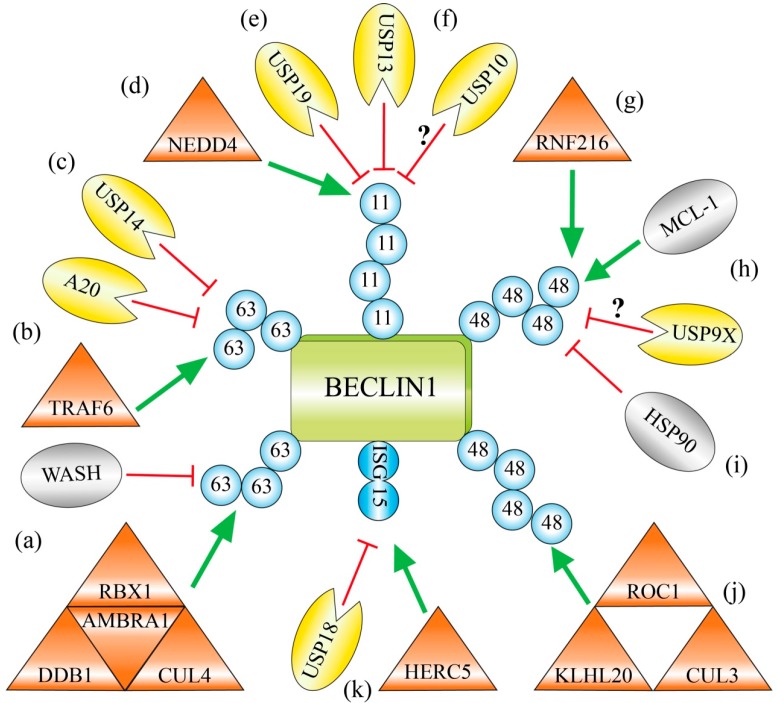
Regulation of BECLIN 1 by ubiquitination and ISGylation. Ubiquitin chains linked via Lys63 support the function of BECLIN 1 and autophagy activity. (**a**) BECLIN 1 is polyubiquitinated with Lys63-linked chains by the AMBRA 1-containing CUL4-ligase complex. WASH prevents this ubiquitination by disruption of the BECLIN 1–AMBRA 1 interaction; (**b**) The RING-type ligase TRAF6 polyubiquitinates BECLIN 1 with Lys63-linked ubiquitin chains. It prevents the association of the BECLIN 1-inhibitor BCL-2 and therefore supports autophagy. The deubiquitinating enzyme A20 opposes this process; (**c**) The deubiquitinating enzyme USP14 cleaves Lys63-linked polyubiquitin chains from BECLIN 1 and thereby down-regulates autophagy; (**d**) The HECT-type ligase NEDD4 catalyzes Lys11-linked polyubiquitin chains on BECLIN 1 and primes it for proteasomal degradation; (**e**) The deubiquitinating enzyme USP19 cleaves Lys11-linked ubiquitin chains and stabilizes BECLIN 1; (**f**) The deubiquitinating enzymes USP10 and USP13 deubiquitinate and stabilize BECLIN 1. USP13 can cleave Lys11-linked ubiquitin chains from BECLIN 1, whereas the corresponding specificity of USP10 is not known; (**g**) The TRIAD-type ligase RNF216 attaches Lys48-linked polyubiquitin chains on BECLIN 1 and marks it for proteasomal degradation; (**h**) BECLIN 1 and the BCL-2 family member MCL-1 induce their destabilization reciprocally. Both compete for the interaction to the deubiquitinating enzyme USP9X, which stabilizes its corresponding binding partner; (**i**) The interaction of BECLIN 1 to the chaperone HSP90 prevents the formation Lys48-linked polyubiquitin chains on BECLIN 1 and protects it against proteasomal degradation; (**j**) The KLHL20-containg CUL3-ligase complex attaches Lys48-linked polyubiquitin chains on BECLIN 1 and triggers it degradation by the 26S proteasome; (**k**) The ubiquitin-like protein ISG15 is attached by the E3 enzyme HERC5 to BECLIN 1. It blocks the lysine residues that are targets for Lys63-linked polyubiquitin chains and therefore inhibits BECLIN 1 function and autophagy. This process is reversed by the deISGylating enzyme USP18. [Shape annotation: triangle = E3 enzyme; pie = deubiquitinating enzyme; oval: other factors.]

The AMBRA 1-containing RBX1/CUL4-ligase complex catalyzes the formation of a Lys63-linked polyubiquitin chain, which is attached to Lys437 of BECLIN 1 (Figure 2a) [67]. The described ubiquitination event has a positive impact on starvation-induced autophagy, as demonstrated in mouse embryonic fibroblasts (MEFs). In cells that harbor an ubiquitination-resistant variant of BECLIN 1, the interaction of BECLIN 1 with VPS34 is weaker and the activity of VPS34 is reduced. The Lys63-linked ubiquitin chain might possibly function as a scaffold that triggers the assembly further factors that are required for the progression of autophagy. Therefore, the ubiquitination by the AMBRA 1-containing CUL4 ligase complex has been reported to be important for autophagy under these conditions [67].

The interaction between BECLIN 1 and AMBRA 1 can be blocked by WASH (*Wiskott–Aldrich syndrome protein and SCAR Homologue*). WASH is a member of the WASP (*Wiskott-Aldrich syndrome protein*) family [73] and has a functional role in endosome sorting [74]. The additional inhibitory function during autophagy is based on the concept that WASH prevents the interaction of BECLIN 1 with the AMBRA 1-containing ligase complex and therefore blocks the Lys63-linked ubiquitination of BECLIN 1 [67].

AMBRA 1 itself is also regulated by ubiquitination events. It is interesting to note that one of these ubiquitination cascades is triggered by its interaction with WASH [75]. WASH recruits the RING-type E3 ligase RNF2 (*ring finger protein 2/Ring1b*), which ubiquitinates the residue Lys45 within AMBRA 1 with a Lys48-linked polyubiquitin chain. This modification primes AMBRA 1 for the degradation by the 26S proteasome and finally results in the down-regulation of autophagy [75,76].

The second described mechanism to down-regulate AMBRA 1 by ubiquitination surprisingly involves CUL4 [77]. This study describes time-dependent steps from the onset to the termination of autophagy. CUL4 binds to AMBRA 1 under fed conditions in order to limit basal autophagy. The phosphorylation of AMBRA by ULK1 leads to a dissociation from CUL4 and enhanced autophagy activity during the first hours of starvation. After prolonged starvation, AMBRA 1 associates again with the CUL4 ligase complex. This results in the Lys48-linked polyubiquitination and rapid proteasomal degradation of AMBRA 1, which leads to the down-regulation of autophagy activation and therefore finally to the termination of the autophagy mechanism [35,78,79].

How the described functional roles of CUL4 as an activator [67] or terminator [77] of autophagy can be integrated in one unified model will require further work.

### 2.2. Lys63-Linked Polyubiquitination and Activation of BECLIN 1 by TRAF6 during Phagocytosis

Macrophages exhibit Toll-like receptors (TLRs) at the extracellular side of their plasma membrane. TLRs have a central role in innate immunity, because they can recognize conserved molecular patterns found in pathogens. TLR4 recognizes bacterial lipopolysaccharides (LPS) and subsequently initiates a signaling cascade, which induces phagocytotic uptake as well as autophagic degradation of the pathogen [80,81]. The intracellular TLR4-adaptor proteins MyD88 and TRIF function as a direct link to PI3K-III signaling because they can interact with BECLIN 1 [82]. This results in a disruption of the interaction between BECLIN 1 and its inhibitor BCL-2 and enables the induction of autophagy [82].

Another way to promote the disruption of the binding of BCL-2 to BECLIN 1 is depending on ubiquitination events. The RING-type E3 ligase TRAF6 (*tumor necrosis factor-associated factor 6*) modifies the residue Lys117 within the BH3 domain of BECLIN 1 with a Lys63-linked ubiquitin chain (Figure 2b) [83]. The ubiquitin chain prevents interaction of BCL-2 with the BH3 domain of BECLIN 1 and therefore enables the BECLIN 1-dependent induction of autophagy [83,84]. The deubiquitinating enzyme A20 hydrolyzes the peptide-bond between BECLIN 1 and the ubiquitin chain. This enables again the binding of BCL-2 to the BH3 domain and therefore suppresses the induction of autophagy [83].

It is interesting to note that the interaction of BECLIN 1 with BCL-2 is regulated in a similar manner by phosphorylation of the BH3 domain of BECLIN 1 [31]. The kinase DAPK (*death associated protein kinase*) can phosphorylate Thr119 within the BH3 domain, resulting in a block of BCL-2 binding and an increase in autophagy [85]. TRAF6 predominantly ubiquitinates substrates that are phosphorylated [86]. Therefore, it will be of interest whether the phosphorylation within the BH3 domain may prime BECLIN 1 for the ubiquitination by TRAF6.

The interaction between BECLIN 1 and BCL-2 can also be prevented when BCL-2 is phosphorylated and unable to bind to the BH3 domain. This phosphorylation is catalyzed by JNK1 (*c-Jun N-terminal kinase 1*) [87]. JNK1 itself is activated by being phosphorylated by the kinase TAK1 (*transforming growth factor β-activated kinase 1*). TAK1 is activated by Lys63-linked polyubiquitination, which is catalyzed by TRAF6 [88,89]. Therefore, TRAF6 can down-regulate the amount of BCL-2 that can associate with BECLIN 1 in two principle ways: TRAF6 can have a direct influence by ubiquitinating the BCL-2-binding motif in BECLIN 1 or it can have an indirect influence by supporting the JNK-dependent phosphorylation of the BECLIN 1-binding motif in BCL-2 [31,83,87].

TRAF6 controls autophagy and BECLIN 1 also via additional pathways. TRAF6 can associate with the autophagic scaffold protein ATG9, which is an important regulatory target of kinase ULK1 [90], resulting in the activation of JNK1 via an unknown mechanism [91]. Moreover, TRAF6 can ubiquitinate ULK1 with a Lys63-linked polyubiquitin chain [92]. This ubiquitination depends on AMBRA 1, which seems to bridge the interaction of TRAF6 and ULK1 and therefore might function as a substrate adaptor like in the context of the Rbx1/Cul4-ligase complex during the ubiquitination of BECLIN 1 [67] (see Section 2.1). The ubiquitinated ULK1 initiates several early steps in the induction of autophagy, including the phosphorylation of BECLIN 1 at Ser14, which results in a higher PI3K-III complex activity [93].

These studies show that TRAF6 is involved in different reactions of the autophagy mechanism and that these steps are often functionally interconnected.

### 2.3. USP14 Suppresses Autophagy by Cleaving Lys63-Ubiquitin Chains from BECLIN 1

USP14 (*ubiquitin-specific protease 14*) is deubiquitinating enzyme that localizes to the cytosol and the 26S proteasome [94]. It is involved in the negative regulation of the proteasome-system via the detachment of Lys48-linked ubiquitin chains as well as in the negative regulation of autophagy by suppressing Lys63-linked chains [95,96]. Recent work demonstrates that the central role of USP14 in the regulation of autophagy lies in its function as deubiquitinase of Lys63-ubiquitin chains attached to BECLIN 1 [97] (Figure 2c). USP14 is activated via AKT-mediated phosphorylation [96]. Moreover, USP14 is a central module in the AKT-dependent signaling, because it is required for the AKT-mediated inhibition of autophagy [97]. The deubiquitination of BECLIN 1 by USP14 inhibits the PtdIns3P production of VPS34. USP14 acts specifically on the Lys63-ubiquitin chains of BECLIN 1 and does not target Lys48-linked chains on BECLIN 1.

The expression of USP14 is increased in a variety of cancers [98]. These studies make it possible to develop USP14 inhibitors that promote autophagy and UPS as a potential treatment in neurodegenerative diseases and cancer.

### 2.4. Lys11-Linked Polyubiquitination and Degradation of BECLIN 1 by NEDD4

The HECT-type ligase [99] NEDD4 (*neural precursor cell-expressed developmentally down-regulated protein 4*) regulates the stability of BECLIN 1 in HeLa cells [100]. The interaction between NEDD4 and BECLIN 1 is required for the formation of Lys11-linked polyubiquitin chains on BECLIN 1 (Figure 2d) and its subsequent degradation in the 26S proteasome. This proteasomal disposal influences the steady-state level of BECLIN 1 and is enhanced when the expression of VPS34 is down-regulated by siRNA treatment [100]. This fosters the assumption that mainly free BECLIN 1 molecules are substrates of NEDD4 in the context of a possible quality control mechanism. It seems that basal BECLIN 1 protein levels have to be tightly regulated. A low cellular concentration of BECLIN 1 is often associated with the occurrence of cancers [101,102], while the overexpression of BECLIN 1 in certain cancers is correlated with a more aggressive clinical behavior and prolonged survival of a subset of tumor cells, most likely by promoting autophagy and thereby preventing apoptosis [103,104].

NEDD4 can influence BECLIN 1 and autophagy also in other ways. NEDD4 has been found to be part of the LC3-interaction map of basal autophagy [105]. This systematic RNAi screen demonstrated that the down-regulation of NEDD4 results in an increase of the steady-state level of the membrane-bound LC3-II after the inhibition of the V-type H(+)-ATPase via Bafilomycin A1 treatment. The results suggest that NEDD4 functions in the down-regulation of basal autophagy [105].

A recent publication suggests a supportive function of NEDD4. At least the experimental down-regulation of NEDD4 inhibits starvation induced autophagy [106], be it directly or indirectly. The interaction of NEDD4 to LC3 stimulates the E3 ligase activity of NEDD4 and ubiquitinated LC3 species can be detected. However, future work will clarify whether this ubiquitination represents an enhancing or self-limiting effect on starvation induced autophagy [106,107].

NEDD4 can inhibit virus-induced autophagy [108]. The Japanese encephalitis virus (JEV) causes the most prevalent viral encephalitis in Asia. In general, autophagy can help to destroy intracellular viral particles. However, virus-induced autophagy was suppressed by an elevated level of NEDD4. This makes NEDD4 a potential drug target, because the experimental down-regulation of NEDD4 can enhance autophagy and therefore decreased the amount of viral particles [108].

NEDD4 functions as a negative regulator of the tumor suppressor p53 [109]. The protein level of p53 is regulated by the proto-oncogenic RING-type ligase MDM2 (*mouse double minute 2*) [110]. The turnover of MDM2 depends on its auto-ubiquitination. NEDD4 stabilizes the MDM2 by attaching Lys63-linked chains to it, which then results in a higher degradation rate of p53 via MDM2-catalyzed ubiquitination [109]. This mechanism could also be of relevance for the function of BECLIN 1. The stability of both BECLIN 1 and p53 is supposed to be regulated reciprocally, which involves a direct interaction between BECLIN 1 and p53, including p53-induced ubiquitination of BECLIN 1 by an unknown E3 ligase [111,112]. Therefore, it will be of interest to see if NEDD4 can regulate BECLIN 1 stability also via this indirect way and thereby influence the dynamic interplay of BECLIN 1 and p53 in particular and the balance between autophagy and apoptosis induction in general [113,114].

In summary, the present data suggest that NEDD4 might possibly act as a negative regulator of the BECLIN 1-p53 axis, because it can target both proteins. However, as both proteins have a context-dependent, reciprocal mode of balancing the basal protective functions of both apoptosis and autophagy, NEDD4 might act as a fine-tuning modulator of both processes.

### 2.5. Deubiquitination of Lys11-Linked Polyubiquitin Chains and Stabilization of BECLIN 1 by USP19 and USP13

USP19 is a tail-anchored deubiquitinase that localizes to the endoplasmic reticulum, where it plays a role in the unfolded protein response (UPS) as well as in the hypoxia pathway [115,116]. Recently, a dual role of USP19 in macroautophagy and antiviral immune responses was discovered [117].

Macroautophagy is supported by USP19. The Lys11-linked ubiquitin chain attached to the lysine residue 437 of BECLIN 1 is removed by USP19 [117] (Figure 2e). USP19 acts specifically on Lys11-linked chains and not on other linkages. As a consequence, BECLIN 1 is stabilized and protected against proteasomal degradation. The Lys11-linked chain-dependent turnover of BECLIN 1 is blocked by the mutation of lysine 437 of Beclin1 or the activity mutant of USP19 [117]. 

Several autophagy-related proteins have autophagy-independent functions in immunity [118,119]. USP19 negatively regulates type I interferon (IFN) signaling pathways. It does so by blocking the RIG-1 (*retinoic acid inducible gene-1*)—MAVS (*mitochondrial antiviral-signaling protein*) interaction in a BECLIN 1-dependent manner. The USP19-stabilized BECLIN 1 binds to MAVS and blocks the interaction to RIG-1. Therefore, the USP19-BECLIN 1 axis stimulates macroautophagy and inhibits the activation of type I interferon signaling [117].

The lysine residue 437 seems to be the main site for the attachment of the Lys11-linked ubiquitin chain on BECLIN 1. However, also other lysines can be modified in this manner. Their deubiqutination is carried out by USP13 in USP19-depleted cells, suggesting a partial redundant role for USP13 in this process [117].

### 2.6. Chemical Inhibition of the Deubiquitinating Enzymes USP10 and USP13 Causes Ubiquitination and Degradation of BECLIN 1

Spautin-1 (*specific and potent autophag*y *inhibitor-1*) is a chemical compound (IUPAC name: 6-fluoro-*N*-[(4-fluorophenyl)methyl]quinazolin-4-amine) that is able to block autophagy [120]. The treatment of mouse embryo fibroblasts (MEFs) with Spautin-1 results in ubiquitination and degradation of BECLIN 1 as well as of VPS34, VPS15 and ATG14L [120]. This results in a significant reduction of PtdIns3P production and leads to an inhibition of autophagy [120].

Spautin-1 inhibits the activity of the deubiquitinating enzymes USP13 and USP10. Moreover, it blocks the direct interaction of USP13 to BECLIN 1 (Figure 2f). Therefore, these findings indicate that USP13 and USP10 protect BECLIN 1 against ubiquitination and degradation [120]. 

The identity of the responsible E3 enzyme and the linkage of the ubiquitin chain are not known. However, because USP13 has been shown to deubiquitinate Lys11-linked polyubiquitin chains on BECLIN 1 [117], it can be speculated that this kind of linkage might also be formed after Spautin-1 treatment. However, this does not exclude the possibility that Lys48-linked chains could also play a role after Spautin-1 triggered inhibition of USP10.

It is not clear whether VPS34 and ATG14L are also directly protected by USP10 and USP13 or if their degradation upon Spautin-1 treatment is caused indirectly by a destabilization due to the breakdown of BECLIN 1. In yeast [121] and mammalian cells [122], it was shown that the reduction of the Vps30/BECLIN 1 level causes instability of certain other PI3K-III complex subunits. Therefore, the degradation of ATG14L may represent an indirect effect, which is caused by the instability of BECLIN 1. It is interesting to note that the knock-down of VPS34 and BECLIN 1 also causes the degradation of USP10 and USP13, which indicates the existence of a regulatory feedback loop [120].

An additional possible link of USP10 to autophagy is based on the finding that USP10 can be co-isolated with ULK1 [123]. ULK1 is a central protein kinase that is involved in the induction of autophagy. Among other targets, ULK1 phosphorylates also BECLIN 1 [93,124] and AMBRA 1 [46,92] in order to initiate autophagy. However, a direct link of a possible USP10-dependent deubiquitination and stabilization of ULK1 and the ULK1-catalyzed phosphorylation of BECLIN 1 and AMBRA 1 has not been demonstrated yet.

Moreover, USP10 has been identified as a deubiquitinating enzyme for p53 [125]. As described before (Section 2.4), the interaction of p53 and BECLIN 1 has been reported to regulate the cellular decision on the induction of autophagy or, alternatively, apoptosis in embryonal carcinoma cells [111].

The relevance of the USP10/USP13-based regulation was supported by further studies. It was demonstrated that the combination of Spautin-1 with other drugs has an enhancing therapeutic effect in the treatment of chronic myeloid leukemia and ovarian cancer [126,127].

### 2.7. RNF216 Catalyzes Lys48-Linked Polyubiquitination of BECLIN 1 and Marks it for Degradation

Toll-like receptors (TLRs) serve as sensors for the detection of specific molecular patterns on various pathogens in the context of innate immunity. TLRs activate signaling events are linked to the induction of autophagy pathways like xenophagy [128].

The E3 ligase RNF216 (*ring finger protein 216*) has been identified as a regulator of innate immunity. It is also called TRIAD3A, because it is a TRIAD-type E3 ligase, which harbors a motif consisting of a RING1, IBR (*in between RING*) and RING2. Therefore this E3 family is called RBR (*RING-Between RING-RING*) or TRIAD (*two RING fingers and a DRIL [double RING finger linked]*) [129]. RNF216 ubiquitinates several TLRs such as TLR4 and TLR9. The target proteins are degraded and therefore TLR signaling is down-regulated [130,131,132].

RNF216 can negatively regulate autophagy in macrophages by ubiquitinating BECLIN 1 [133] (Figure 2g). This ubiquitination can be induced by TLR4 activation as well as starvation conditions. RNF216 binds BECLIN 1 via its Triad domain and modifies it with Lys48-linked polyubiquitin chains. RNF216 is not able to catalyze Lys29 or Lys63-linked chains on BECLIN 1. The polyubiquitinated BECLIN 1 species are degraded in the 26S proteasome and autophagy is inhibited [133].

Experiments using mice infected with *Listeria monocytogenes* demonstrated a reciprocal correlation of RNF216 levels compared to autophagy activity and bacterial growth. Therefore, RNF216 modulates TLR-mediated antimicrobial responses via the control of autophagy [133].

The general interplay of RNF216- and BECLIN 1-dependent processes is also important in the context of tumorigenesis. RNF216 is up-regulated in human colorectal cancer (CRC) cells. RNF216 promotes CRC cell proliferation and migration in association with an enhancement of proteasomal degradation of BECLIN 1. Vice versa, the knockdown of RNF216 increased autophagic activity and limited CRC cell proliferation and migration. Therefore, RNF216 is regarded as a potential biomarker and novel therapeutic target for the inhibition of CRC development and progression [134].

### 2.8. USP9X Governs the Reciprocal Ubiquitin-Based Interplay of BECLIN 1 and MCL-1

MCL-1 (*myeloid cell leukemia sequence 1*) is a BCL-2 family member. High levels of MCL-1 have been shown to correlate with the occurrence of several types of tumors [135,136,137]. The role in tumorigenesis has been associated with its anti-apoptotic functions. However, the role in the regulation of mitochondrial bioenergetics and morphogenesis [138] or RAS signaling [139] may add to this functional implication in tumor-promotion. A direct link to starvation-induced autophagy is based on the interaction of MCL-1 with BECLIN 1 [140]. MCL-1 and BECLIN 1 regulate each other in a reciprocal manner, which is governed by USP9X [140] (Figure 2h).

The deubiquitinase USP9X stabilizes both MCL-1 [140,141,142] and BECLIN 1 [140] and protects them against polyubiquitiation and proteasomal degradation. MCL-1 is ubiquitinated with a Lys48-linked chain by the E3 enzyme MUEL25 [143,144]. MUEL5 does not ubiquitinate BECLIN 1 and the corresponding E3 enzyme as well as polyubiquitin chain type are unknown in the case of BECLIN 1 [140].

BECLIN 1 and MCL-1 induce their destabilization reciprocally. This is the case because both compete for the binding to USP9X [140]. This correlation is also found in patient-derived melanoma cells and tissue samples. The BECLIN 1 levels decrease and the MCL-1 level increase in an inter-dependent manner during melanoma progression [140]. Therefore, the indirectly supported, USP9X-mediated degradation of MCL-1 can be regarded as a novel autophagy-independent, oncosuppressive function of BECLIN 1 [145]. Moreover, this dual regulation of MCL-1 and BECLIN 1 adds another layer to the complex functions of USP9X, which can act as potential oncogen or tumor supressor [146].

### 2.9. The Chaperone HSP90 Protects BECLIN 1 against Lys48-Linked Polyubiquitination and Degradation

HSP90 (*heat shock protein 90*) is a molecular chaperone that assists protein folding [147]. It has been demonstrated that HSP90 can be inhibited by Geldanamycin, which is a benzoquinone ansamycin antibiotic [148]. Interestingly enough, Geldanamycin also blocks the interaction between BECLIN 1 and HSP90. This results in the Lys48-linked polyubiquitination of BECLIN 1 (Figure 2i) [149]. The Lys48-linked polyubiquitination primes BECLIN 1 for degradation by the 26S proteasome and thereby reduces the BECLIN 1 protein level and autophagy activity [149]. The E3 ligase that targets BECLIN 1 in absence of the interaction to HSP90 is unknown. However, several other HSP90 binding partners have been reported to be substrates of the CUL5-based E3 ligase complex [150,151]. However, whether the CUL5-complex also ubiquitinates BECLIN 1 is not known.

The proto-oncogene p53 was demonstrated to counteract the ATPase activity of HSP90 [152,153]. This could be of potential interest, because the described interaction of p53 with BECLIN 1 (see Section 2.4) in embryonic carcinoma cells has been found to induce Lys48-linked polyubiquitination and proteasomal degradation of BECLIN 1 [111].

In addition to BECLIN 1, the central autophagy factors ATG7 and ULK1 are stabilized by HSP90 [154,155], which makes HSP90 an important positive regulator for autophagy. Because tumor cells are protected by a functional autophagy system, HSP90 has become a molecular target of anti-cancer therapies [156,157,158].

### 2.10. Limitation of Autophagy by KLHL20-Catalyzed Lys48-Linked Ubiquitination of BECLIN 1, ULK1 and VPS34

Autophagy is a self-limiting process, because prolonged failure of termination would lead to degradation of cells and tissue [159,160]. This termination is mediated via the degradation of autophagic key factors. The complex ligase CUL3-KLHL20 is an important factor involved in this process [161]. CUL3 and the RING-type ligase subunit ROC1 (*regulator of cullins 1*) associate with the substrate adaptor KLHL20 (*kelch-like 20*) [162] and down-regulates a subset of autophagy-relevant proteins that act early in autophagy. This includes the autophosphorylated form of the serine/threonine kinase ULK1 as well as VPS34 and BECLIN 1 (Figure 2j). The KLHL20-based ligase complex attaches Lys48-linked ubiquitin chains on the substrates and thereby marks them for proteasomal degradation [163]. Moreover, other proteins are affected indirectly by the activity of the KLHL20-based ligase complex. The degradation of ULK1 induces the instability ATG13 and the degradation of BECLIN 1 induces the instability of ATG14L.

It is interesting to note, that the KLHL20 ligase complex is not the only E3 targeting ULK1. It has been demonstrated that the HECT-type ligase NEDD4L can ubiquitinate at ULK1, which results in the proteasomal degradation of ULK1 and down-regulation of autophagy [164]. It is not known whether NEDD4L can also target BECLIN 1.

The regulated termination of autophagy via this pathway seems to be important. Depletion of KLHL20 or replacement of ULK1 with a KLHL20-binding mutant enhances cell death induced by prolonged starvation [163]. KLHL20-KO mice show that ablation of KLHL20 enhances diabetes-associated muscle atrophy [163]. Thus, while the induction of autophagy is important for the preservation of the structure and function of the muscle by removing damaged cell components [165,166], a regulated termination of autophagy via the degradation of key factors is required for cellular homeostasis [163,167,168].

### 2.11. Competition with the Ubiquitin-Like ISG15 Interferes with Polyubiquitin Chain Formation on BECLIN 1

Posttranslational modifications with ubiquitin-like molecules play a role in the regulation of PI3K-III complex activity, as shown by the stimulating effect of the SUMOylation of VPS34 [169]. Recent work has demonstrated that BECLIN 1 can be modified with the ubiquitin-like protein ISG15 (*interferon-stimulated gene 15*) [170] (Figure 2k).

In general, ISG15 is involved in anti-viral pathways and ISG15-deficient mice are highly susceptible for viral infections like herpes or influenza [170,171]. In general, ISG15 is an important type I interferon (IFN) effector system. It contains two ubiquitin-like domains [172]. Similar to ubiquitin, it is also conjugated via a cascade of E1/E2/E3 enzymes, with HERC5 (*HECT domain and RCC1-like domain containing protein 5*) as the central E3 ligase [173]. This process is called ISGylation, while the detachment is called deISGylation. The deISGylation is catalyzed by USP18 (also called UBP42) [174,175].

Type I IFN can induce the expression of ISG15, which results in ISGylation of BECLIN 1 at several lysine residues [170]. The ISGylation competes with the Lys63-linked ubiquitination of BECLIN 1. Because the Lys63-linked polyubiquitination functions as positive regulator of PI3K-III complex activity (Section 2.1, Section 2.2 and Section 2.3), the induction and progression of autophagy is down-regulated when the Lys63-chains cannot be formed [170]. This process is opposed via the deISGylation by USP18. Even though the immunity-related functions of autophagy seems to have a special focus on the uptake of bacteria, viral infections may often be supported by the autophagy machinery. This is in line with reports describing that certain viruses can exploit the autophagy machinery such as autophagosomes and use them for multiplication [176,177]. This option is suppressed by type I IFN-mediated ISGylation of BECLIN 1, which suppresses autophagy by blocking the formation of Lys63-linked ubiquitin chains on BECLIN 1 [170].

Future work will have to correlate the autophagy-suppressing function of ISG15 in the context of xenophagy with the apoptosis-supporting function as well as with the potential to influence chemosensitivity in esophageal cancer cells [178].

## 3. Conclusions

The phosphorylated signaling lipid PtdIns3P has a central function in three the topologically related membrane involution processes endocytosis, cytokinesis and autophagy. The core components of the PtdIns3P-generating PI3K-III complex are the lipid kinase VPS34, the membrane-anchoring protein kinase VPS15 as well as the adaptor protein BECLIN 1. Because most of the regulatory factors bind via BECLIN 1, it is reasonable that BECLIN 1 is a highly regulated protein. The biological activity of BECLIN 1 can be transiently altered via posttranslational modifications like phosphorylation, acetylation, ISGylation and ubiquitination (Table 1).

BECLIN 1 is one of the few known proteins to be regulated not just by one or two but by several ubiquitination cascades, as described in this manuscript. The known E3 ligases and DUB systems concerning the ubiquitination of BECLIN 1 have been identified in single-focused studies. Therefore, it will be of interest to combine the single outcomes of the studies in order to generate a coherent picture of the mechanistic network that regulates BECLIN 1 function. One open question is, whether there is a synergistic effect of the autophagy-supporting ligases that produce Lys63-linked chains. At least for TRAF6 it is known that it supports autophagy directly by ubiqutinating BECLIN 1 and ULK1 as well as indirectly by the modification of proteins that induce phosphorylation of BECLIN 1 and BCL-2 in order to prevent the association of BECLIN 1 with its inhibitor. Furthermore, it will be important to know if TRAF6 and the CUL4-complex both are active at the same time in the same cells during the early stages of autophagy or if each of them is specific for certain tissues or autophagy-inducing conditions. Similarly, it would be important to know whether the Lys11-linked ubiqutitn chains (NEDD4; USP19/USP13) and Lys48-linked ubiquitin chains (RNF216, CUL3-complex, HSP90) are correlated or context-dependent. It could be possible that some may be more relevant for BECLIN 1 steady state levels and basic autophagy activity, while others might be predominantly required for the concerted and rapid degradation of BECLIN 1 and other key autophagy factors for the termination of highly induced autophagy pathways.

Moreover, it will be even more important to extend the current knowledge concerning the cross-talk between ubiquitination and phosphorylation. Therefore, it is tempting to speculate that the corresponding kinases and E3 ligases are involved in a time-dependent, cascade-like regulation of autophagy during its initiation, execution and termination.

The elucidation of the systemic mechanisms of E3 enzymes involved in the regulation of BECLIN 1 will important to understand the complex interplay within the regulatory network of PtdIns3P-signaling in the occurrence of infections, neurodegenerative diseases and cancer.

## Figures and Tables

**Table 1:** Factors involved in the regulation of BECLIN 1 by ubiquitin and the ubiquitin-like ISG15. The corresponding stimulating (+) or inhibiting (−) effect on autophagy is listed. Information that is not known or that has not directly been investigated is marked with a [?]. We refer to the text for more detailed information about the factors listed in this summary table.

Name	Type	Target Lys in BECLIN 1	Modification of BECLIN 1	Effect on Modification	Effect on BECLIN 1	Effect on Autophagy
CUL4-complex	E3 (CUL)	Lys437	PolyUb (Lys63)	Synthesis	Strong VPS34 binding	+
WASH	WASP	Lys437	PolyUb (Lys63)	Inhibition	Weak VPS34 binding	−
TRAF6	E3 (RING)	Lys117	PolyUb (Lys63)	Synthesis	no BCL-2 binding	+
A20	DUB	Lys117	PolyUb (Lys63)	Hydrolysis	BCL-2 binding	−
USP14	DUB	[?]	PolyUb (Lys63)	Hydrolysis	weak ATG14L/UVRAG binding	+
NEDD4	E3 (HECT)	[?]	PolyUb (Lys11)	Synthesis	Degradation	− [?]
USP19	DUB	Lys437	PolyUb (Lys11)	Hydrolysis	Stabilisation	+
USP13	DUB	[?]	PolyUb (Lys11)	Hydrolysis	Stabilisation	+
USP10	DUB	[?]	PolyUb (Lys11) [?]	Hydrolysis	Stabilisation	+
RNF216	E3 (RBR)	[?]	PolyUb (Lys48)	Synthesis	Degradation	−
MCL-1	BCL-2 Family	[?]	PolyUb (Lys[?])	Induction	Degradation	−
USP9X	DUB	[?]	PolyUb (Lys[?])	Hydrolysis	Stabilisation	+
HSP90	HSP	[?]	PolyUb (Lys48)	Inhibition	Stabilisation	+
CUL3-complex	E3 (CUL)	[?]	PolyUb (Lys48)	Synthesis	Degradation	−
HERC5	E3 (for ISG)	Lys117/263/265/266	ISGylation	Synthesis	Weak VPS34 binding [?]	−
			PolyUb (Lys63)	Inhibition		
USP18	DUB (for ISG)	Lys117/263/265/266	ISGylation	Hydrolysis	Strong VPS34 binding [?]	+
			PolyUb (Lys63)	no Inhibition		
